# The Dangers of Taxis

**Published:** 1902-12-13

**Authors:** 


### THE DANGERS OF TAXIS.

Mr. McGavin communicated a case of strangulated inguinal hernia in which the employment of taxis had led to reduction *en masse*, and pointed out that the history of the case emphasised what had been repeatedly insisted on by many authorities, namely, that it was unwise to attempt to reduce a strangulated hernia by taxis, and that when persisted in repeatedly such treatment was quite unjustifiable. In the discussion it was pointed out that taxis was used in a good many cases, and that it was probably only the unsuccessful ones that one heard about. Mr. Bowlby said that although reduction *en masse* occurred in a certain number of cases in which taxis was applied by the patient and his friends, he had never yet seen a case in which it had followed taxis as applied in the hospital. He thought it would lead to considerable loss of life if taxis were quite abandoned, for even if the mortality from operation were only 10 per cent., that from taxis, if applied within 24 hours, would not be nearly as much.

There is much to be said, however, for the contention of the author of the paper; and it is at any rate a matter of some interest that all the papers read at this meeting should have pointed to the advantage of operating in the open, guided by sight rather than by faith.

